# Network pharmacology and molecular docking to explore the mechanism of Sheng Xue Bao mixture against iron deficiency anemia

**DOI:** 10.1097/MD.0000000000035012

**Published:** 2023-09-15

**Authors:** Yun Wang, Huang Qinqin, Haixia Wang, Hongxu Zhang, Xinhua Zhang, Weiguo Liu, Zhenhua Xiang, Yuming Gu

**Affiliations:** a Department of Traditional Chinese Medicine, Affiliated Hospital of Weifang Medical University, School of Clinical Medicine, Weifang Medical University, Weifang, China; b Weifang Medical University, Weifang, China; c Clinical Research Center, Affiliated Hospital of Weifang Medical University, Weifang, China.

**Keywords:** iron deficiency anemia, network pharmacology, Sheng Xue Bao mixture

## Abstract

Based on network pharmacology and molecular docking, we investigated the mechanism of action of Sheng Xue Bao mixture (SXBM) in treating iron deficiency anemia (IDA). We screened the HERB and traditional Chinese medicine systems pharmacology database and analysis platform databases to identify the active ingredients and targets of SXBM. The targets associated with “iron deficiency anemia” were collected from GeneCards, TTD, and OMIM databases. A component-target interaction network was constructed using Cytoscape 3.8.2. The protein-protein interaction network of candidate targets was generated using the STRING database and visualized with Cytoscape 3.8.2 software. Core modules obtained from clustering analysis were subjected to Gene Ontology and Kyoto encyclopedia of genes and genomes enrichment analysis. Finally, molecular docking validation of key targets and active components was performed using Autodock Vina software. A total of 174 active components and 111 genes were identified as potential active components and targets for IDA treatment, including quercetin, kaempferol, luteolin, beta-sitosterol, and other flavonoids as main active components. Gene Ontology enrichment analysis show that interleaved genes are enriched in 2328 biological processes, 71 cellular component expression processes, and 157 molecular function processes. Kyoto encyclopedia of genes and genomes analysis mainly envolved Prostate cancer, Hepatitis B, Kaposi sarcoma-associated herpesvirus infection, Endocrine resistance, Lipid and atherosclerosis, Central carbon metabolism in cancer, Human cytomegalovirus infection and HIF-1 signaling pathway. STAT3, SRC, PIK3R1, and GRB2 were selected as core targets. The molecular docking results demonstrated strong interactions between key components and their respective target proteins. Network pharmacological analysis suggested that SXBM could treat IDA by regulating various biological processes and related signaling pathways. It laid the foundation for further elucidating the molecular mechanism of SXBM treatment of IDA.

## 1. Introduction

Iron deficiency anemia (IDA) is a form of microcytic hypochromic anemia which is mainly caused by a deficiency of iron in the body, resulting in impaired hemoglobin synthesis in the cells.^[1]^ It is one of the most common anemias in the world and has a negative impact on the daily lives of more than 2 billion people worldwide.^[2]^ Due to iron deficiency, the activity of many enzymes is reduced, which affects cell metabolism, which has certain blood system manifestations on the immune function, behavior, and development of the human body, gastrointestinal tract, skin and mucous membranes, and nervous system. For example, patients with iron-deficiency anemia usually have features such as fatigue, palpitations, white eyelids and lips, and a yellow complexion. These phenomena are mainly caused by long-term iron deficiency in the body.^[3]^ However, from the perspective of microscopic pathology, it is manifested in low levels of red blood cells and hemoglobin backlog in blood, low transferrin in serum, and a relatively low iron level.^[4]^ Currently, the international treatment of iron deficiency the anemia is mainly based on the supplementation of inorganic iron sulphate or organic iron complex to improve the absorption of iron in the body.^[5]^ Nevertheless, these treatment options are prone to a range of side effects such as constipation, diarrhea and weight loss. As well as the use of various iron-fortified foods, which may not only have the above-mentioned adverse effects, but also have low absorption and utilization of inorganic salts by the body.^[6]^

The research on “iron-deficiency anemia in traditional Chinese medicine has a history of more than 2000 years. Modern scholars believe that iron deficiency anemia belongs to the category of “blood deficiency.”^[7]^ Chinese medicine considered the liver to be the main function of producing blood and the kidney to store essence, the inherent inner foundation of the body. As stated in “Plain Questions • Six Sections Zangxiang Lun Chapter 9”: “The liver... it is born in the tendons to generate blood and qi.” Therefore, in the treatment of iron-deficiency anemia, it is mainly treated from the perspective of nourishing the kidney and nourishing the essence, nourishing the blood, and regulating the liver. The modern Chinese patent medicine Sheng Xue Bao mixture (SXBM) meets the above requirements and has the effect of nourishing the liver and kidney, nourishing qi, and producing blood. Based on this, the modern proprietary Chinese medicine SXBM has the effect of nourishing the liver and kidneys, nourishing qi and promoting blood production, thus meeting the above requirements. Our previous meta-analysis study has confirmed that SXBM is indeed effective in the treatment of IDA.^[8]^ But the molecular mechanism of traditional Chinese medicine in the treatment of iron deficiency anemia still lacks modern scientific research. Recently, network pharmacology has gained worldwide prominence. It has multi-directional cross-integration features that enable comprehensive network analysis of herbal medicines and their compound prescriptions, and it provides a systems-level comprehension of the pathogenesis of diseases. Due to the integrity and systematic nature of its research strategy, it has obvious advantages and potential in elucidating its mechanism of action and explaining the composition of the formulation, in line with the overall concept of TCM evidence-based treatment. In this study, the pharmacological and molecular basis of the SXBM for the treatment of iron deficiency anemia was analyzed from the perspective of network pharmacology, and a “drug effect-element-target continuum” correlation network was established for a multi-group study of the SXBM. The results were further validated using molecular docking study techniques to provide a basis for the ensuing basic experimental studies and clinical trials.

## 2. Data and methods

### 2.1. Screening for active ingredients and target genes

SXBM includes Heshouwu (Polygoni Multiflori Radix), Nvzhenzi (Fructus Ligustri Lucidi), Sangshen (mulberry fruit), Mohanlian (Eclipta Prostata), Baishao (Radix Paeoniae Alba), Gouji (Cibotium barometz), and Huangqi (Astragalus). The drugs in the SXBM were sequentially input into the traditional Chinese medicine systems pharmacology database and analysis platform (TCMSP) database for retrieval, and the active ingredients and targets were screened according to the setting of OB value ≥ 30% and drug-like properties value ≥ 0.18. The OB value refers to the speed at which the drug is absorbed into the human body, drug-like properties refers to the structural similarity of herbal ingredients to a known drug. If the drug was not found in the TCMSP database (http://tcmspw.com/tcmsp.php), the ingredients retrieved from the herb database (http://herb.ac.cn/) were used as potential active ingredients. Swiss target prediction is a similarity-based principle that utilizes reverse screening of a database designed to predict the most likely protein targets of small molecules, targets were obtained through the Swiss target prediction database.

### 2.2. Screening of disease targets

In the previously mentioned meta-analysis, SXBM was found to be effective in treating iron deficiency anemia with significant therapeutic effects. In order to further understand the mechanism of action of SXBM for the treatment of iron deficiency anemia, we have to first identify the target of iron deficiency anemia. We identified the keyword as “iron deficiency anemia” in OMIM (https://omim.org/), Genecards (https://www.genecards.org/), TTD database (http://db.idrblab.net/ttd/) to find the target of iron deficiency anemia-related diseases.

### 2.3. Looking for the target of SXBM in the treatment of IDA

The drug targets in the SXBM obtained through the above methods and the disease targets for IDA obtained in the above 3 databases were overlapped by the Venny 2.1 online mapping platform to predict the SXBM Potential active components of the mixture for the treatment of iron deficiency anemia.

### 2.4. Construction of PPI network map and screening of core genes

The drug-disease common targets were entered into the String database (https://string-db.org/cgi/input.pl) to build a proteinprotein interaction （PPI) protein network map. In a PPI protein network diagram, each node represents a protein encoded by an active ingredient or gene, and the edges in the network diagram represent protein-protein relationships or protein-target relationships. It is to be noted that the protein type “Homo sapiens” should be selected for searching, and the Interaction between minimum threshold should be set to 0.9 to obtain the network relationship data of the target interaction. In order to further screen core genes, the obtained network relationship data of target interaction can be imported into Cytoscop 3.8.2, using the NetworkAnalyzer tool for in-depth topology analysis, and the MCODE module for gene cluster analysis and core target screening.

### 2.5. GO enrichment analysis and Kyoto encyclopedia of genes and genomes (KEGG) pathway analysis

**Figure 1.**
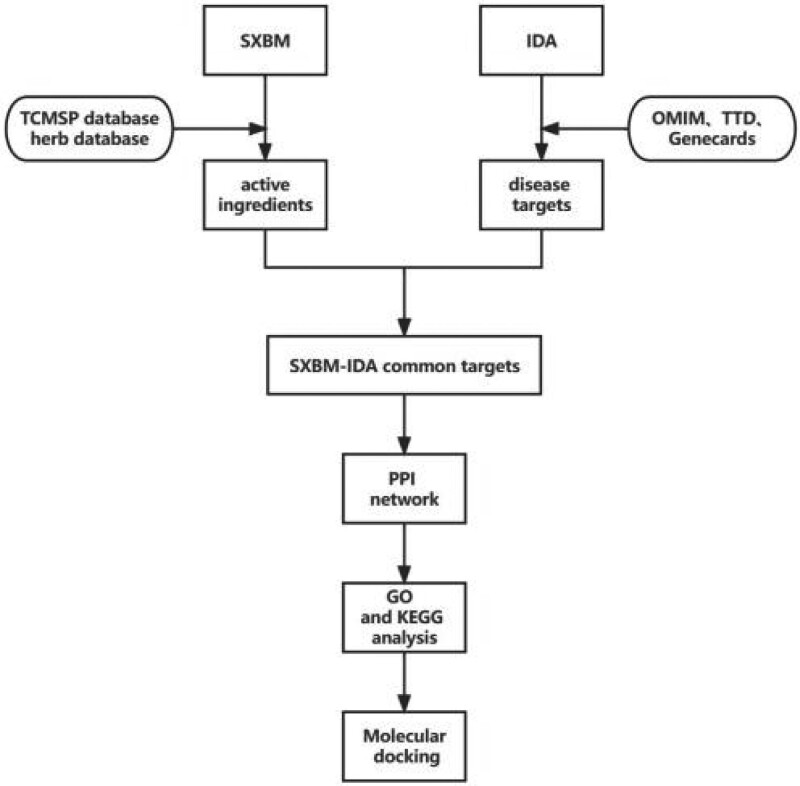
Flow chart of the study.

Through the comparison and analysis of target pathways, we analyzed the core genes screened from SXBM and their roles in signaling pathways. To explore its function to realize the function of this gene, we used the R software-based Bioconductor bioinformatics package to achieve Gene Ontology (GO) enrichment and KEGG pathway analysis. And visualize the results with Rstudio. Our flow chart of the study is shown as Figure 1.

### 2.6. Molecular docking of core components of SXBM with key target genes

The above methods are all predictions of the target genes for the SXBM treatment of IDA. In the following, we will use the molecular docking method to verify whether the above predictions are accurate. The interaction between the core component of the SXBM and the key target is analyzed using the molecular docking technique. We downloaded SXBM-IDA Target PPI network in the RCSB PDB database (https://rcsb.org) and changed it into a pdb format structure file, and used pymol software to convert the above-mentioned targets. The 4 targets were subjected to hydrogenation, water removal, solvent molecule removal, unwanted ligand removal, and homodimer retention of only single chains, and then exported to pdbqt format files using autodocktools software. Download the top 4 active ingredients in terms of degree value in the Pub Chem database (https://pubchem.ncbi.nlm.nih.gov/). The sdf format structure files of the components is exported to pdb format files after hydrogenation by pymol software, and they are then exported to pdbqt format files after defining Torsion using autolock tools software. Open the pdbqt format file of the receptor protein and ligand small molecule in the autolocktools software, generate the docking box and adjust the parameters, run autodock vina to dock the 4 targets with the 4 active components of SXBM, and analyze the docking results obtained. The binding strength and activity are evaluated based on the binding energy and the number of generated hydrogen bonds between the receptor protein and the ligand small molecule. The results were visualized using pymol and the Discovery Studio software.

## 3. Result

### 3.1. The analysis of the potential drug components

The active ingredients of Astragalus, Wolfberry, Eclipta, Mulberry, and Ligustrum lacidum can be found in the TCMSP database, and the active ingredients of Gouji and Polygonum multiflorum can be queried in the herb database, a total of 174 potential active ingredients was obtained after deduplication (Table 1). Among them, there are 45 kinds of active ingredients in Lycium barbarum, 20 active ingredients in Astragalus, 13 active ingredients in Ligustrum lacidum, 10 active ingredients in Eclipta chinensis, 6 active ingredients in Mulberry, 34 active ingredients in Gouji, 57 active ingredients in Polygonum multiflorum potential. After that, 820 drug targets were deduplicate and screened from the Swiss target prediction database, of which 423 targets were predicted for Lycium barbarum, 429 targets for Astragalus, 231 targets for Ligustrum lucidum, 226 targets for Eclipta chinensis, and mulberry 126 targets, 430 targets for Gouji, and 561 targets for Polygonum multiflorum.

**Table 1:** Operative ingredients of SXBM.

Mol ID	Molecule name	OB(%)	DL
MOL001323	Sitosterol alpha1	43.28	0.78
MOL003578	Cycloartenol	38.69	0.78
MOL001494	Mandenol	42	0.19
MOL001495	Ethyl linolenate	46.1	0.2
MOL001979	LAN	42.12	0.75
MOL000449	Stigmasterol	43.83	0.76
MOL000358	Beta-sitosterol	36.91	0.75
MOL005406	Atropine	45.97	0.19
MOL005438	Campesterol	37.58	0.71
MOL006209	Cyanin	47.42	0.76
MOL007449	24-methylidenelophenol	44.19	0.75
MOL008173	Daucosterol_qt	36.91	0.75
MOL008400	Glycitein	50.48	0.24
MOL010234	Delta-carotene	31.8	0.55
MOL000953	CLR	37.87	0.68
MOL009604	14b-pregnane	34.78	0.34
MOL009612	(24R)-4alpha-Methyl-24-ethylcholesta-7,25-dien-3beta-ylacetate	46.36	0.84
MOL009615	24-Methylenecycloartan-3beta,21-diol	37.32	0.8
MOL009617	24-ethylcholest-22-enol	37.09	0.75
MOL009618	24-ethylcholesta-5,22-dienol	43.83	0.76

DL = drug-like properties, CLR = Cholestrol, LAN = Lanosterol, OB = Oral bioavailability.

**Figure 2.**
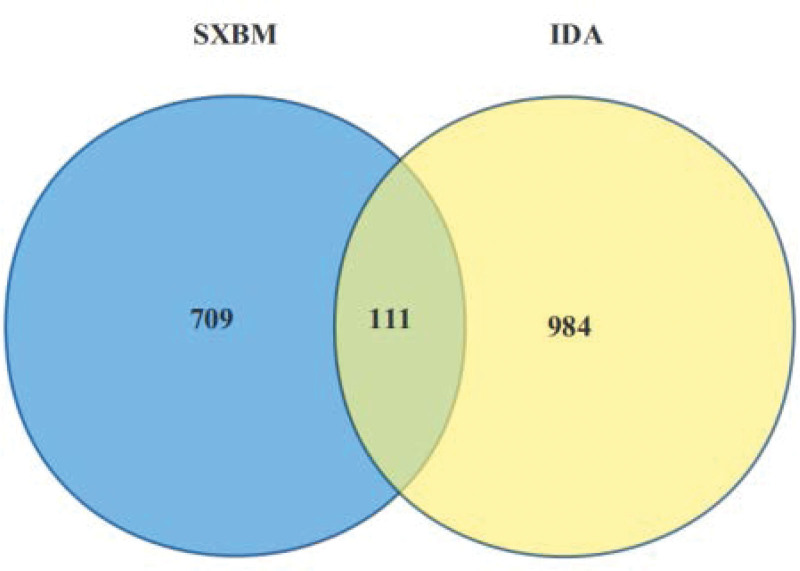
Venn diagram of common target genes for Sheng Xue Bao mixture (SXBM) and iron deficiency anemia (IDA).

A total of 841 IDA-related genes were obtained from the Genecards database, 1104 from the OMIM database, and 1 from the TTD database. After deduplication, a total of 1095 IDA-related genes was found. The screened drug targets and disease targets were superimposed to obtain 111 common targets (Fig. 2), which will serve as potential targets for SXBM in the treatment of IDA.

### 3.2. Key target PPI network

**Figure 3.**
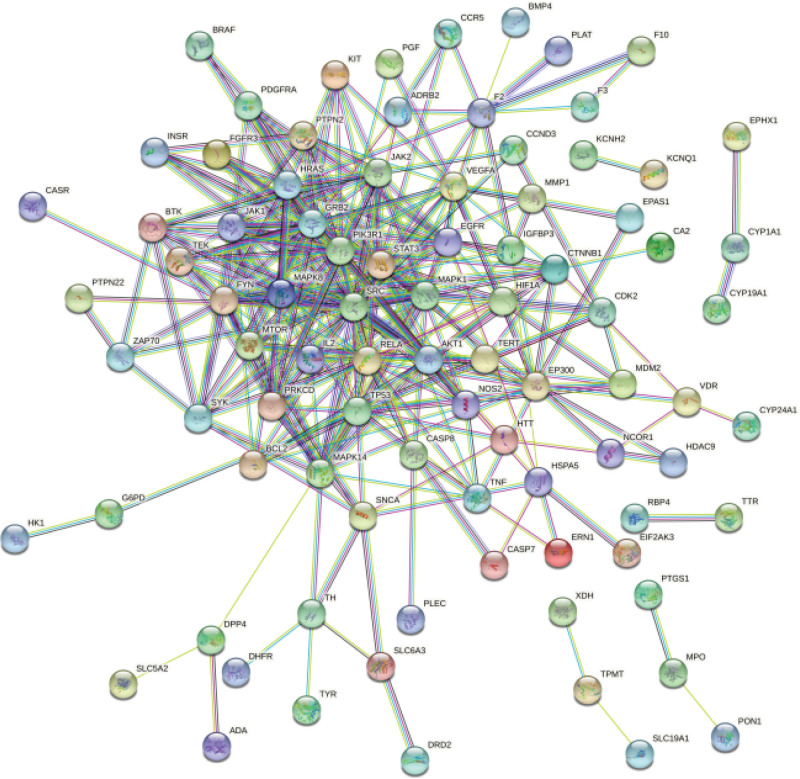
PPI network diagram from String.

**Figure 4.**
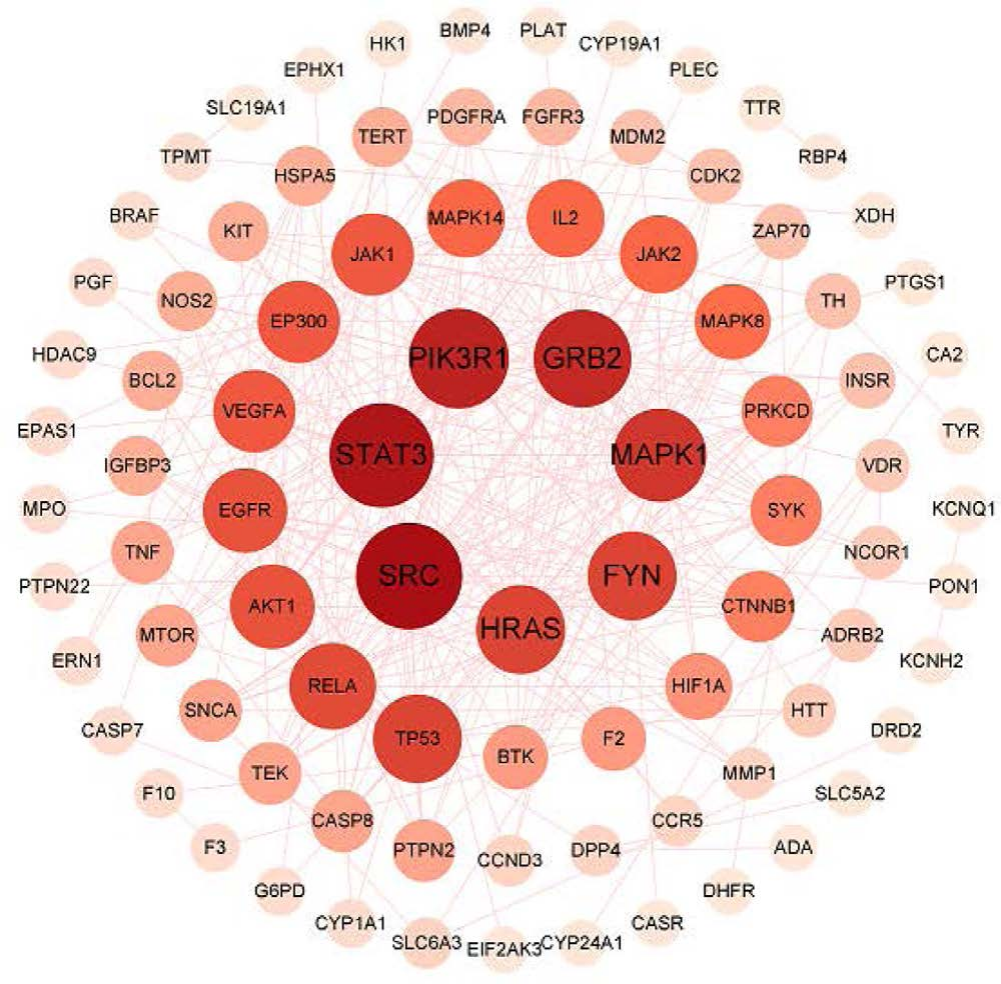
PPI network diagram from Cytoscape.

**Figure 5.**
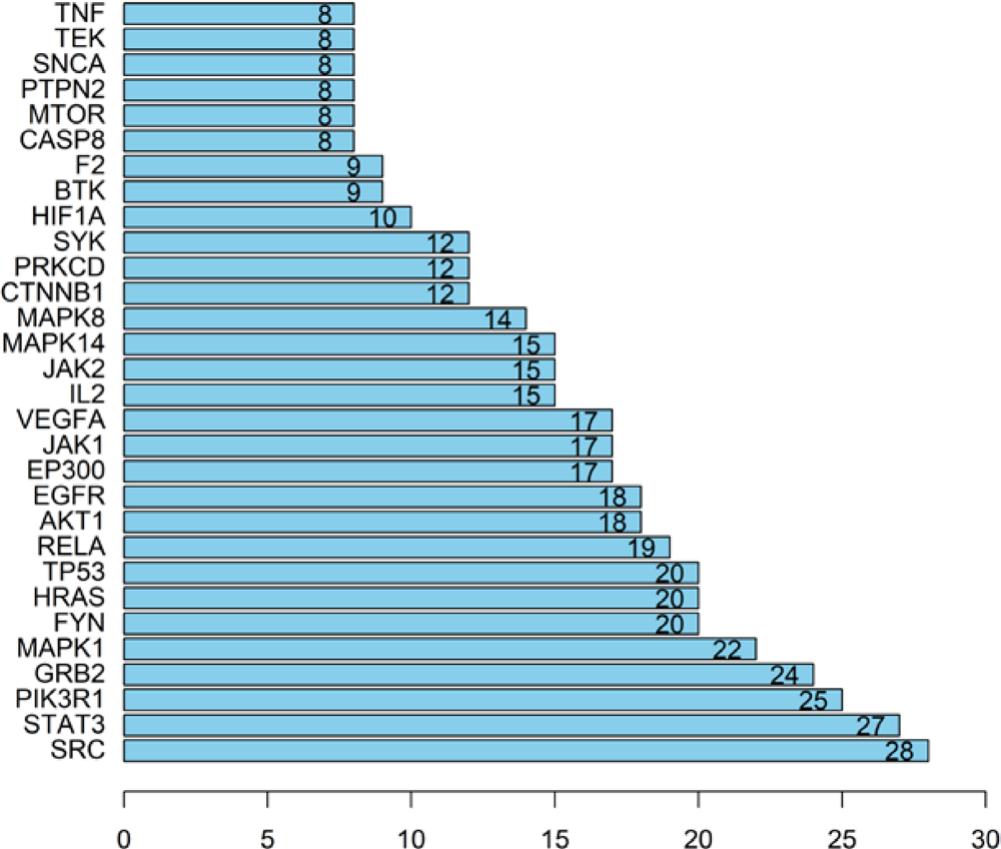
30 key targets determined in the PPI network.

After adding 111 common targets to the String database for retrieval, removing isolated nodes can construct a PPI map of protein interactions (Fig. 3). We imported it into Cytoscape software and obtain a new PPI diagram (Fig. 4). It can be seen from the PPI diagram that a total of 311 edges have been established, which means that there are 311 interactions between proteins. The thickness of the edge represents the relationship between the proteins. The thicker the edge, the stronger the relationship among the proteins. It can be read from Figure 4, which are SRC, STAT3, PIK3R1, GRB2, MAPK1, FYN, and HRAS genes in order of degree value. We further used Network Analyzer to perform topological analysis on the network graph (Fig. 5), where the X coordinate represents the Degree value, that is, the number of associations between the component and the target. The larger the Degree value, the more important the function of the gene in the organism. It can be ulteriorly verified from Figure 4 that the genes which play the important roles in the treatment of IDA by SXBM.

### 3.3. Core component screening

**Figure 6.**
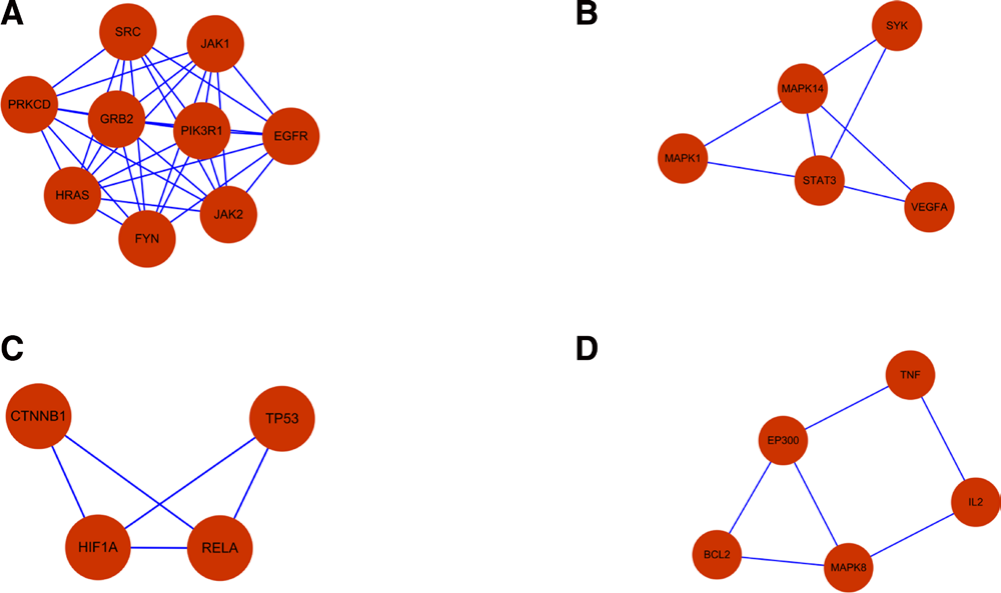
Major gene clusters for treating iron deficiency anemia (IDA) in Sheng Xue Bao mixture (SXBM).

The genes that have been screened in the PPI network map are subjected to topological analysis by Cytoscape 3.8.2 software, and the key components of SXBM can be obtained. Using the MCODE module, the analysis of gene clusters and the screening of core targets can be performed. From this, 4 gene clusters (Fig. 6) and 3 core genes can be obtained. The core genes are GRB2, STAT3, and HIF1A respectively.

### 3.4. GO enrichment analysis

**Figure 7.**
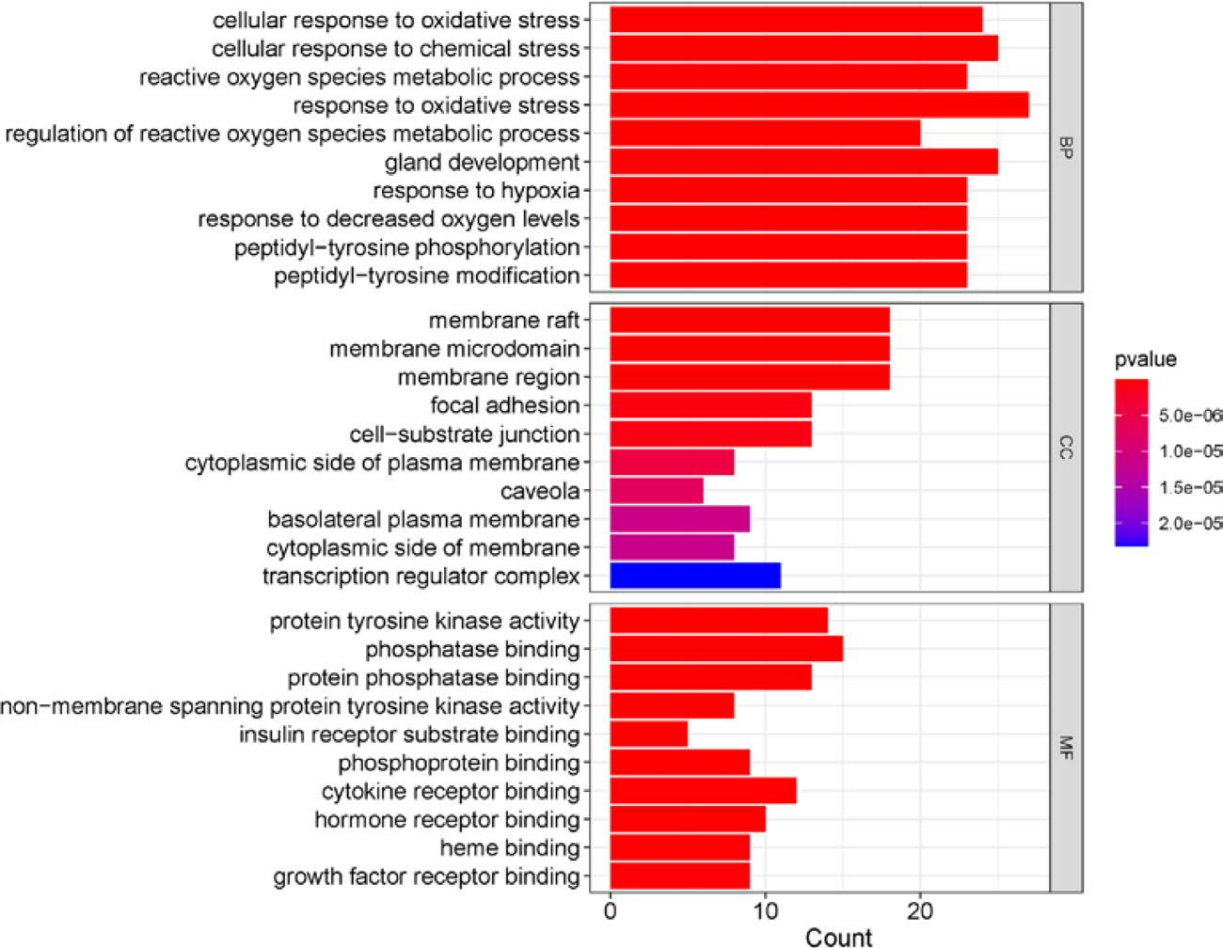
GO enrichment analysis.

We further performed GO enrichment analysis in order to more clearly understand the role of target genes in the treatment process. If the Bioconductor software package is set to *P* value < .05 and *Q* value < 0.05, 111 common targets will be analyzed by R language GO to obtain 3 parts: biological process, cellular components, and molecular functions. The results of the GO enrichment analysis show that interleaved genes are enriched in 2328 biological processes; the intersection genes were enriched in 71 cellular component expression processes; Intersecting genes are enriched in 157 processes related to molecular function. Select the top 10 pathways by *P* value for each part to draw a bar graph (Fig. 7). Then it can be seen from the bar chart that the biological process terms of top10 mainly include cellular response to oxidative stress, cellular response to chemical stress, reactive oxygen species metabolic process, response to oxidative stress, gland development, response to hypoxia, response to decreased oxygen levels, peptidyl-tyrosine phosphorylation and peptidyl-tyrosine modification pathways. The top molecular functions terms mainly include protein tyrosine kinase activity, phosphatase binding, protein phosphatase binding, non-membrane spanning protein tyrosine kinase activity, insulin receptor substrate binding, phosphoprotein binding, cytokine receptor binding, hormone receptor binding, heme binding and growth factor receptor binding. The enriched cellular components terms evolved membrane raft, membrane microdomain, membrane region, focal adhesion, cell-substrate junction, cytoplasmic side of plasma membrane, caveola, basolateral plasma membrane, cytoplasmic side of membrane.

### 3.5. KEGG enrichment pathway analysis

**Figure 8.**
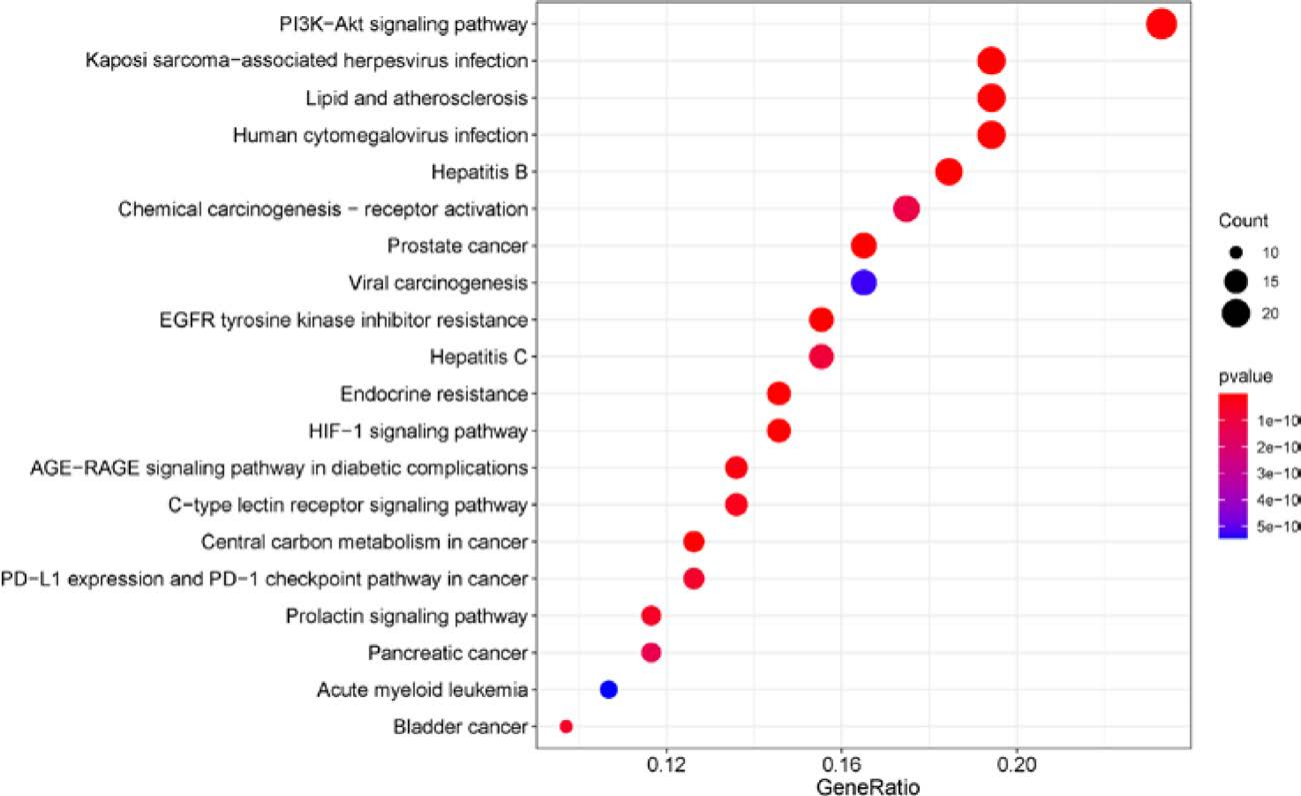
Kyoto encyclopedia of genes and genomes (KEGG) enrichment analysis.

A total of 147 KEGG pathways were obtained from the KEGG enrichment analysis of 111 common targets in the R language. The top 20 pathways by *P* value were selected to draw a bubble chart of KEGG enrichment (Fig. 8). And KEGG analysis mainly envolved Prostate cancer, Hepatitis B, Kaposi sarcoma-associated herpesvirus infection, Endocrine resistance, Lipid and atherosclerosis, Central carbon metabolism in cancer, Human cytomegalovirus infection and HIF-1 signaling pathway, etc.

### 3.6. TCM-component-target-disease network map analysis of SXBM

**Figure 9.**
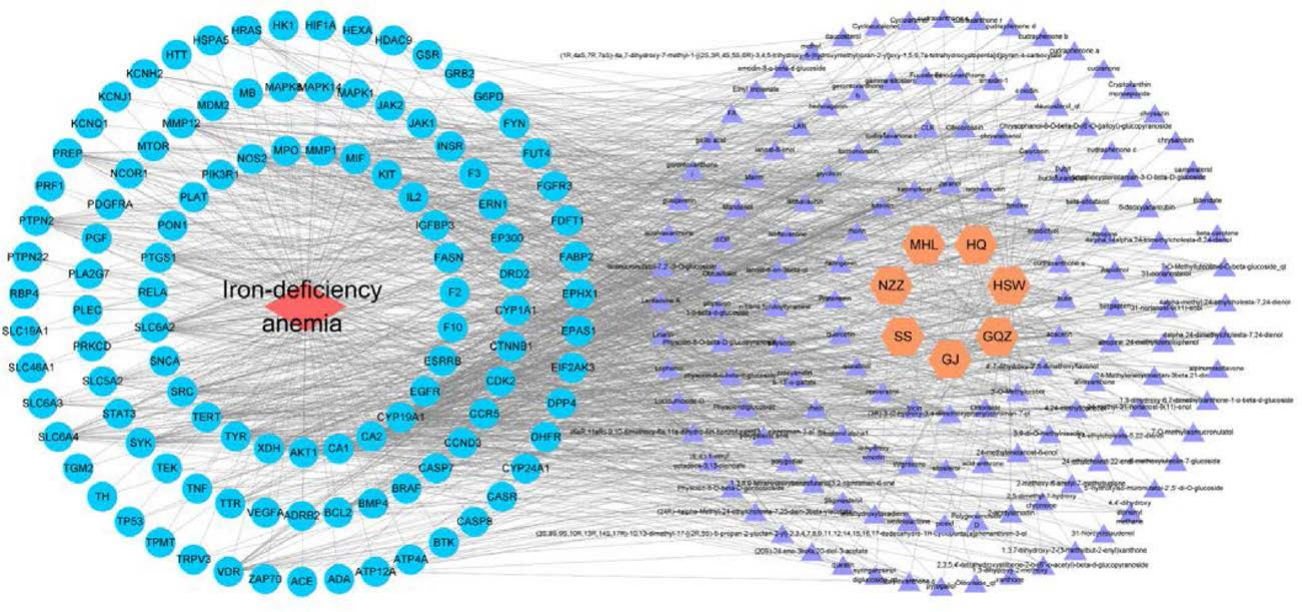
Network of component-targets pathways of Sheng Xue Bao mixture (SXBM) treating iron deficiency anemia (IDA).

Through the traditional Chinese medicine-component-target-disease network diagram, the relationship among components, targets, and pathways in the treatment of IDA by SXBM can be seen more intuitively (Fig. 9). The yellow circles in the figure represent drugs, the purple images represent the 142 active ingredients in the traditional Chinese medicine compound (32 active ingredient targets have no intersection with disease targets and will be deleted), while the blue part represents 111 common targets, and the red diamonds. The squares represent IDA. The network diagram can directly show that SXBM has the characteristics of multi-component and multi-target interaction in the treatment of IDA.

### 3.7. Validation of the relationship between SXBM components and core targets by molecular docking

**Figure 10.**
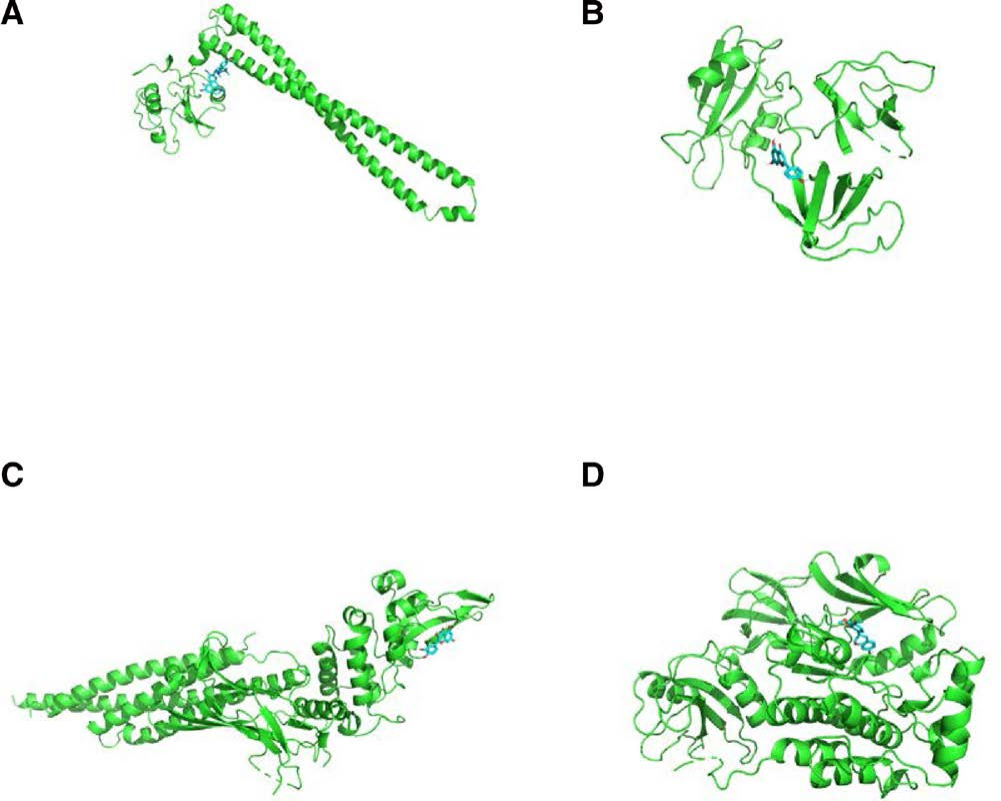
The results of Molecular docking.

Molecular docking was carried out between the targets with the highest degree value in the PPI network and the active components of SXBM. The receptors were: PIK3R1 (PDB: 7myn), GRB2 (PDB: 1gry), STAT3 (PDB: 6NJS), SRC (PDB: 2h8h). The binding strength and activity were evaluated according to the binding energy and the number of generated hydrogen bonds. Since the higher the absolute value of the binding energy during the molecular docking process, the better the affinity between the receptor and the ligand is. In this study, the binding energy ≤ −5.0 kcal/mol was selected as the screening basis for the effective binding of key active ingredients to the core target. The molecular docking results are shown in Table 2. The above docking results were visualized by PyMOL software, and the results were partially shown in Figure 10. The ligand molecule fetidine binds to the protein and forms hydrogen bonds with the amino acid residues SER429, GLN432, and GLN572 of the protein receptor PIK3R1, and the ligand molecule naringenin binds to the protein. The active site of the protein, and forms a hydrogen bond with the amino acid residue VAL213 of the protein receptor GRB2, the ligand molecule quercetin binds to the protein, and forms a hydrogen bond with the amino acid residues GLN644, PRO639, GLN644, LYS658, TYR657 of the protein receptor STAT3. The ligand molecule dimethoxyphenyl binds to the active site of the protein and forms a hydrogen bond with the amino acid residues THR338 and GLU339 of the protein receptor SRC. The result shows that the main active components of SXBM can play a regulatory role through multiple core targets such as PIK3R1, GRB2, STAT3 and so on.

**Table 2:** The docking results of ligand molecules and core target molecules (kcal/mol).

Ligand molecules	Core targets			
	PIK3R1	GRB2	STAT3	SRC
Fetidine	−8.1	−6.3	−5.5	−9.0
Naringenin	−7.6	−6.7	−6.5	−9.2
Quercetin	−7.1	−6.6	−6.2	−8.8
Dimethoxyphenyl	−7.1	−5.9	−5.9	−8.9

## 4. Discussion

IDA is mainly due to insufficient storage or intake of iron in the body. Lack of iron or insufficient utilization of iron can lead to problems in the transport and storage of oxygen, carriage of carbon dioxide, and redox metabolic processes in the body, which can affect human growth and development and even lead to various diseases such as anemia. Iron is one of the vital trace elements in the human body. It is the major ingredient of hemoglobin, myoglobin, cytochromes and some respiratory enzymes. It participates in the transport, exchange, and tissue respiration of oxygen and carbon dioxide in the body.^[9]^

At present, oral iron preparations still cannot achieve the desired curative effect, so some scholars pointed out that the application of traditional Chinese medicine preparations such as SXBM in IDA treatment can promote the absorption of iron preparations and reduce its related adverse reactions.^[10]^ The SXBM is composed of 7 traditional Chinese medicines, including Nvzhenzi, Heshouwu, Sangshen, Mohanlian, Baishao, Huangqi, and Gouji. Among them, Nvzhenzi, Mohanlian, and Sangshen have the function of tonifying the liver and kidney, as well as nourishing the vital energy and the blood. Heshouwu has the effect of nourishing blood, Baishao has the function of tonifying the liver and kidney, as well as nourishing the vital energy and the blood. Gouji is useful for cultivating the kidney. Therefore, the effect of SXBM is mainly to nourish the liver and kidney, nourish qi and consolidate the root, and promote blood and nourish the blood. On this basis, this study takes a look at the pathological mechanism of SXBM in the treatment of IDA to reference its clinical application.

In this paper, a total of 174 components of the SXBM were collected through TCMSP and other databases, mainly including quercetin, kaempferol, luteolin, beta-sitosterol, and other key active components, which constitute multiple common genetic targets with IDA. Quercetin is a flavonoid with anti-inflammatory, anti-viral, anti-allergic and other biological activities.^[11]^ Quercetin has been found to promote the body absorption of ferritin.^[12]^ Studies have shown that kaempferol, which often exists as a variety of different glycosides, can reduce the expression of inflammatory factors such as TNF-ɑ and has anti-inflammatory, antioxidant, immunomodulatory and tumor suppressive effects, among which the antioxidant effect is more pronounced.^[13]^ Another study shows that the ethyl acetate extract of kaempferol has good antioxidant activity, and its mechanism is mainly to enhance the body against anemia by promoting the proliferation, differentiation, and immunity of red blood cells.^[14]^ In addition, the pathways collected by KEGG enrichment mainly include pathways related to cancer, immune system and hematopoietic function. Among them, the HIF-1 pathway is directly related to hematopoietic function. Studies have shown that the HIF pathway can direct the expression of EPO transcripts, hence promoting mRNA production.^[15]^ The PI3K-Akt signaling pathway as a critical signal transmission axon regulating hematopoietic cell survival, growth and the expansion of hematopoietic cells.^[16]^ Finally, molecular docking results showed that STAT3, SRC, PIK3R1, and GRB2 were the core groups in SXBM. Among them, STST3 is mainly involved in mediating the cellular response process of interleukin, LEP and other growth factors, and SRC mainly promotes the formation and transportation of platelets by regulating the formation of megakaryocytes.^[17]^ Studies have shown that GRB2 associates various cytokine signals with the proliferation pathway of HSPC, especially GRB2 associates IL3 signaling with the ERK/MAPK pathway to induce the proliferation and survival of hematopoietic stem cells.^[18,19]^ Activation of cell surface receptors tyrosine kinase growth factor and hormone receptors by PI3Ks translates into downstream AKT activation to regulate cell metabolism, size, differentiation, proliferation, migration and apoptosis.^[20]^ The above core genes were highly matched with the target genes in SXBM, indicating that the target of SXBM was highly correlated with the IDA gene.

This study investigates the mechanism of SXBM for IDA by utilizing network pharmacology. It is known to improve anemia in the body by promoting angiogenesis, anti-apoptosis, and anti-inflammation, providing a theory to define the mechanism of action of SXBM for IDA treatment. Considering the limitations of existing data analysis based on the network and the interaction of drugs in the decoction process, the current results can provide a reference direction for the follow-up research, but the specific results still need further experimental verification.

## Author contributions

**Data curation:** Xinhua Zhang.

**Project administration:** Weiguo Liu.

**Resources:** Hongxu Zhang.

**Supervision:** Haixia Wang.

**Visualization:** Huang Qinqin.

**Writing – original draft:** Yun Wang.

**Writing – review & editing:** Zhenhua Xiang, Yuming Gu.
